# A short history of structure based research on the photocycle of photoactive yellow protein

**DOI:** 10.1063/1.4974172

**Published:** 2017-01-31

**Authors:** Marius Schmidt

**Affiliations:** Physics Department, University of Wisconsin-Milwaukee, 3135 N. Maryland Ave, Milwaukee, Wisconsin 53211, USA

## Abstract

The goals of time-resolved macromolecular crystallography are to extract the molecular structures of the reaction intermediates and the reaction dynamics from time-resolved X-ray data alone. To develop the techniques of time-resolved crystallography, biomolecules with special properties are required. The Photoactive Yellow Protein is the most sparkling of these.

Photoactive Yellow Protein (PYP) was discovered in 1985 by Meyer^1–3^ in cell lysates of a halophilic purple bacterium called *Ectothiorhodospira halophila*, now *Halorhodospira halophila.* PYP is a structural archetype of the PAS (Per-ARNT-Sim) domain superfamily^4^ that is responsible for a vast range of stimuli ranging from light sensing to small ligands.^4,5^ In particular, it was thought of being responsible for the negative phototaxis of *H. halophila*.^6^ Due to the potential biomedical implications of the PAS domain's structural changes, PYP enjoyed major attention by a large number of research groups. Since it can be produced fairly easily and in large amounts,^7^ due to its small molecular weight (14 700 Da) and its interesting physicochemical properties (see below), it quickly became important for structure based dynamics investigations. It was discovered that upon blue light absorption, PYP displays a photocycle^8^ that is based on the *trans* to *cis* isomerization of the central chromophore para-hydroxy-cinnamic acid also called p-coumaric acid (pCA).^9–11^ Conditions to obtain PYP crystals were determined rapidly^12^ and its structure was finally determined in 1995 by Borgstahl and colleagues to 1.4 Å (Ref. 13) (Fig. 1). PYP forms exceptionally well ordered crystals that scatter to sub-Å, atomic resolution.^14–16^

**FIG. 1.**
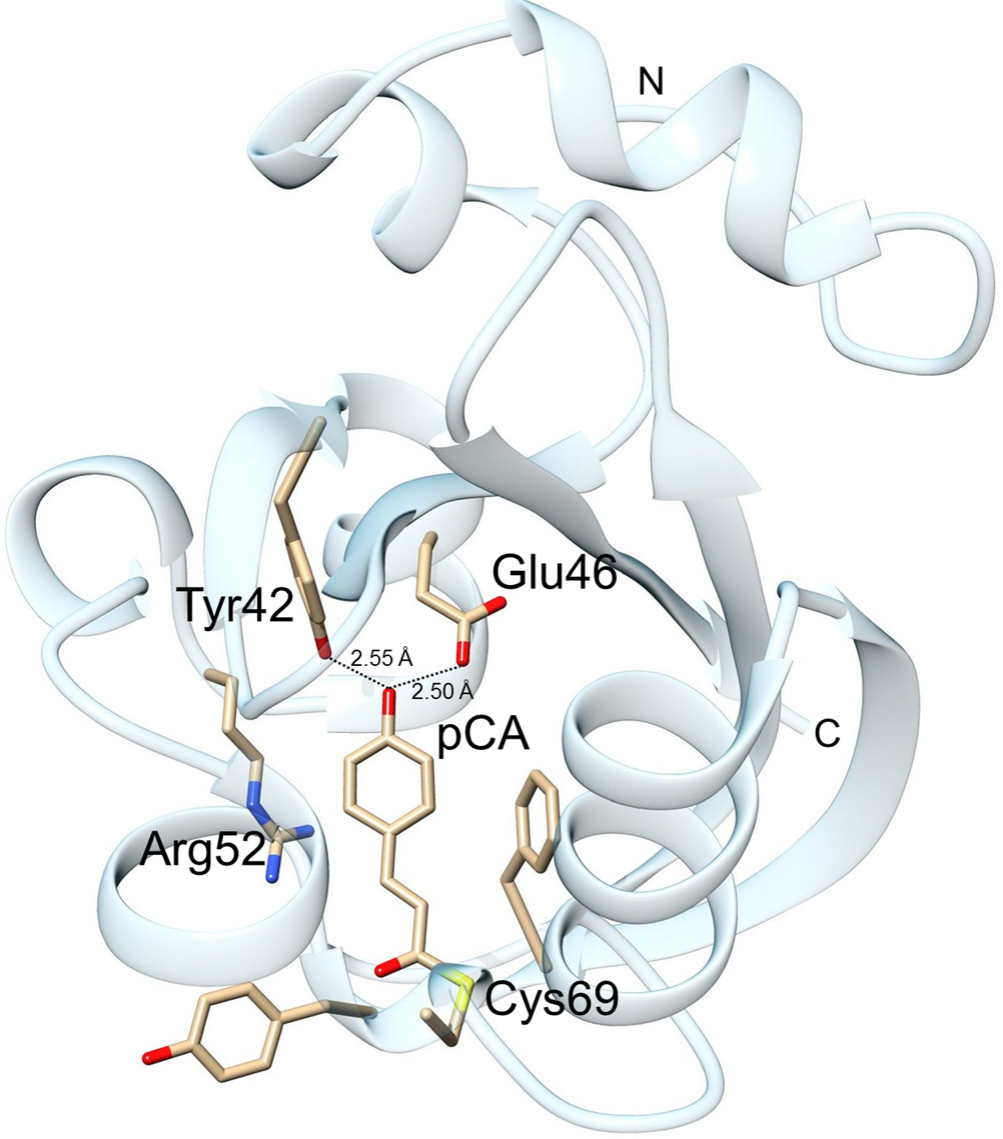
The structure of PYP. The pCA chromophore is attached covalently to Cys69 and forms 2 short hydrogen bonds with Glu46 and Tyr42.^15^

The turn of the millennium was the heyday for time-resolved crystallography with the Laue method, which has been pioneered by Moffat and colleagues.^17,18^ Before any substantial work on PYP could be done, the photocycle was investigated by spectroscopy on faster and faster time scales with ever better time resolution.^19–23^ Already in 1999, it became clear that there are early intermediates that accumulate on the picosecond time scale^20^ and decay through a cascade of other intermediates back to the reference state. The photocycle as established spectroscopically at the end of the millennium is shown in Fig. 2. The interest to determine all structures of the intermediates was enormous. In a first approach, the most stable, longest living intermediate in the photocycle was investigated by producing a photostationary state by exciting PYP crystals with a long blue laser pulse (200 ms, 496.5 nm). The decay of the photostationary state back to the dark state was followed with millisecond time resolved crystallography.^24^ The structure extracted at that time is essentially the structure of the blue shifted intermediate pB as determined later (Fig. 3(c)). In 1998, technology was advanced enough that single pulse Laue exposures with 100 ps time-resolution became feasible.^25^ A single early time point, 1 ns after a laser pulse, was collected on the PYP photocycle.^26^ The interpretation of the difference electron density was difficult, because at 1 ns three intermediates contribute to the photocycle. It took another 15 years until this mixture was finally resolved using a time series on a time range from 100 ps to 10 ns.^2,27^ In the early 2000s, the Laue data collection method was mature and automatic enough to collect entire time series that consisted of multiple time points.^28,29^ This then rose the question how to interpret such a time series. It is fairly straight forward to integrate the difference electron density over the same volume of interest for each and every time point in the time series.^25,28,30,31^ However, the determination of the structures of the intermediates demanded a global analysis of the difference electron density found in all voxels in all difference maps of the time course.^29^ This was finally accomplished by applying a component analysis, the singular value decomposition (SVD), to a series of time-dependent difference maps.^32^ PYP was the driving scientific application.^33–35^ The SVD separates space dependencies into the left singular vectors, and the corresponding time dependencies into the right singular vectors. By interpreting the significant right singular vectors by a kinetic model, the time-independent difference electron densities of the intermediates can be determined by projection using the left singular vectors.^32,36^ A program called SVD4TX^32,37^ was developed (and is available from the author) to perform such a crystallo-kinetic analysis. Various reviews outline the principles of an SVD based analysis of X-ray data.^38,39^ This type of global analysis can provide relaxation times (kinetic phases), the structures of the intermediates, a candidate chemical kinetic mechanism and its associated rate coefficients. In the meantime, static freeze trap experiments were conducted^16^ at cryogenic temperatures, which were suggesting the presence of a mixture of intermediates at early times. There was justified hope that an SVD analysis of a time series of crystallographic data collected on fast time scales would allow for the separation of this mixture at ambient temperatures.

**FIG. 2.**
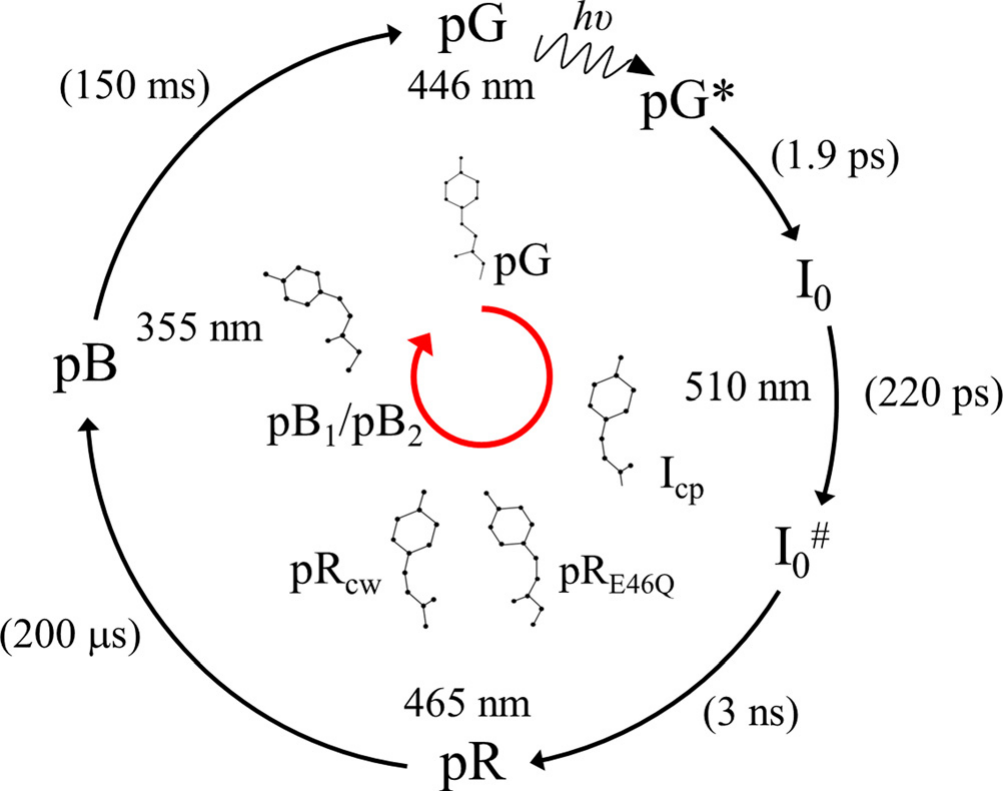
PYP photocycles. Outer cycle: results from spectroscopy established in 1999. Numbers in brackets: relaxation times from global analysis of spectral data. Wavelengths represent absorption maxima of respective states. Structures on the inner, red circle as known in 2005.^24,33,40^ Structures are associated with the spectral states. The structure of I_0_ and those on the fast and ultra-fast time scale (<2 ns) were unknown at this time.

**FIG. 3.**
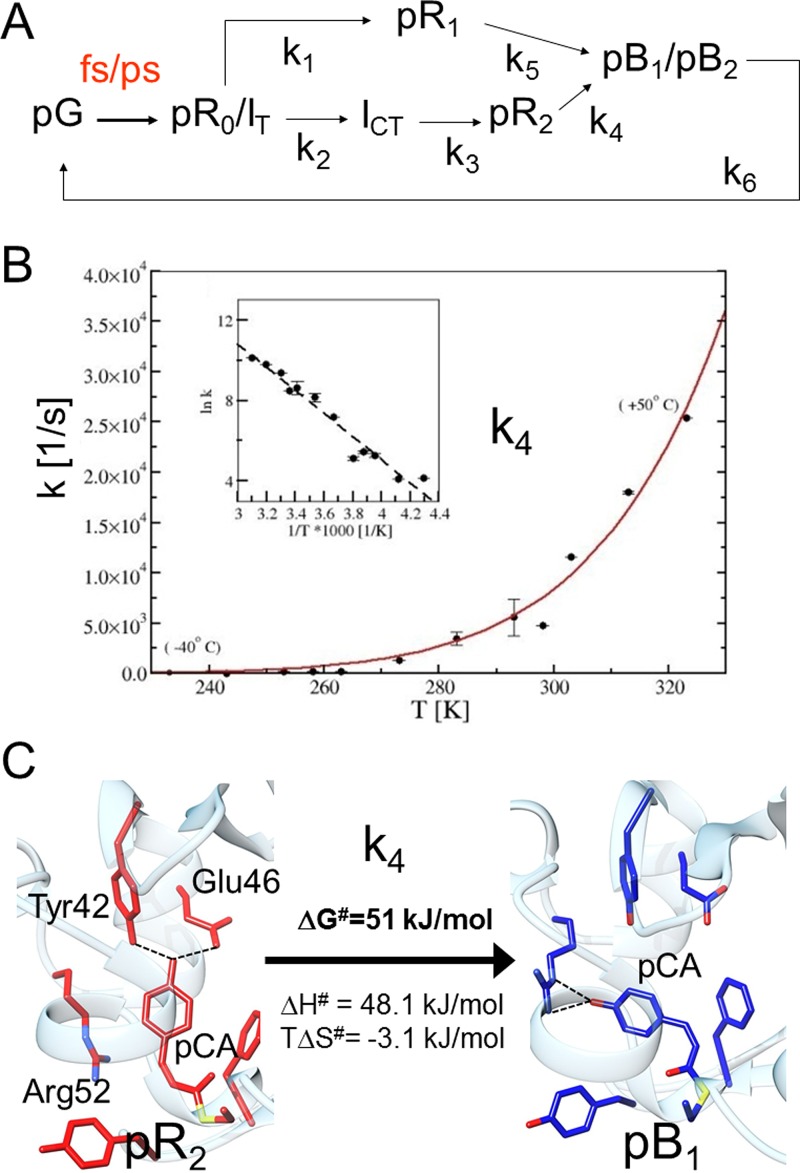
The energetics of the PYP photocycle. (a) Simplified chemical, kinetic mechanism as established by time-resolved crystallography in 2013.^2,27,56^ (b) The temperature dependence of rate coefficient k_4_ as determined by 5D crystallography. Inset: Arrhenius plot. (c) Structural transition caused by k_4_ from pR_2_ to pB_1_. ΔG^#^: free energy difference to the transition state with ΔH^#^ enthalpy and TΔS^#^ entropy (T = 295 K) contribution.

The first time-series of PYP difference maps from 100 ns to 100 ms already revealed interesting details about the difference maps that could be associated with the intermediate states. This time-series was then subject to the SVD. On the early microsecond time scale, up to 100 *μ*s, a mixture of two structures was identified. Evidence from spectroscopy suggests that these intermediate states are populated at the same time delays than the pR spectroscopic state. This mixture persists on approximately the same time scale, and therefore could not be separated by SVD. The two structures that interpret this mixture were refined and denoted pR_cw_ and pR_E46Q_ in 2005^40^ (Fig. 2). A pR_cw_-like structure was first identified at cryogenic temperatures,^16^ and pR_E46Q_ was the single intermediate populated in a time-resolved crystallographic experiment on the PYP E46Q mutant.^41^ pR_cw_ stands for red shifted PYP intermediate where the chromophore tail atoms (C_1_,C_2_,C_3_, C_1′_, see Fig. 4 for the chemical structure of pCA) do not lie in the same plane, and rather adopt a wobbled *cis* configuration. The chromophore head is still bound to Glu46 and Tyr42, as also suggested by spectroscopy. However, in pR_E46Q_, the chromophore is detached from Glu46 and forms one hydrogen bond to Tyr42. Both pR_cw_ and pR_E46Q_ were believed to originate from a single structure, pR_cp_ (cis-planar), that is almost identical to that of pR_cw_. The pR_cp_ structure was also identified earlier at low temperatures.^16^ In pR_cp_, the chromophore tail adopts a planar *cis* configuration. With improved time-resolution, this view was modified slightly (see below). On time scales longer than 100 *μ*s, another intermediate is identified where the chromophore is completely detached from the hydrogen bond network of Tyr42 and Glu46 and forms a hydrogen bond to Arg52^33,40^ (Fig. 3(c)). This structure is reminiscent of the structure of the photostationary state determined already in 1997.^24^ The presence of this pB-like structure already at these early times contradicts the structural interpretation of spectroscopic data at this time delays.^23^ It is now fairly established that a structure with the pCA chromophore detached from Glu46 and Tyr42 accumulates within 100–200 *μ*s. pB stands for blue shifted PYP intermediate. This pB-like structure evolves further to another pB-like intermediate that shows strong difference density features on the N-terminal helix. Accordingly, these pB-like structures were called pB_1_ and pB_2_. It is speculated that the N-terminal helix (see Fig. 1) is involved with signal transduction^40–43^ of this blue light receptor. After pB_2_, the dark state is recovered on a 50 ms time scale. In solution, the dark state recovery is about an order of magnitude slower, suggesting further relaxation and exposure of the chromophore to water that causes the large spectral blue shift. In summary, up to 2007, it was established that there are differences between solution and crystals.^44^ It appears, however, that the structures of the intermediates are largely consistent with their counterparts in solution, especially on faster time scale. An exception maybe a longer lived pB species that is more unfolded in solution.^45–47^ The photocycle as viewed by time-resolved crystallography consisted of pR_cp_, pR_cw_, pR_E46Q_, pB1, pB2, and the reference (ground) state (Fig. 2).

**FIG. 4.**
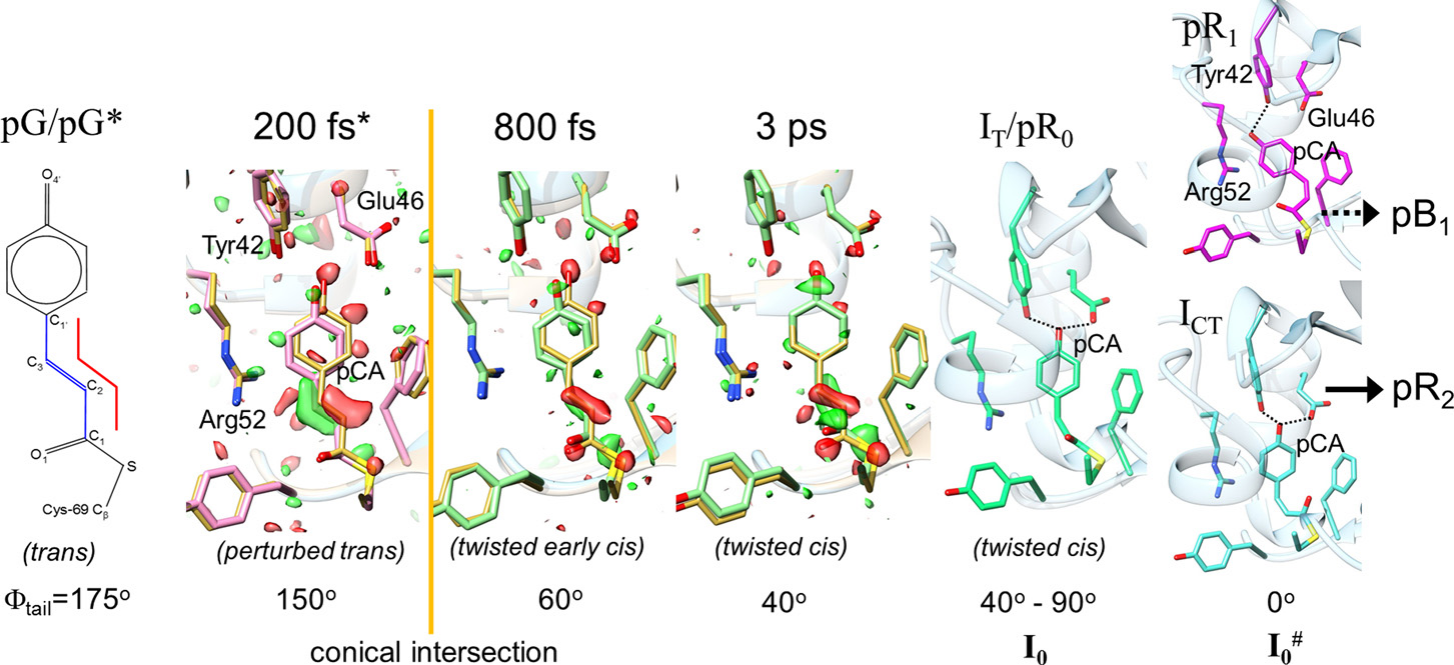
The early part of the PYP photocycle. pG/pG*: chemical structure of the reference structure in the dark and structure of the electronic excited state instantaneously after excitation. Structures in yellow: reference structure as a guide to the eye. Difference electron density in red/green at 200 fs*, 800 fs, and 3 ps. 200 fs*, pink structure: electronic excited state structure, 200 fs after excitation, on the electronic exited state PES. 800 fs, green structure: early electronic ground state structure, 800 fs after excitation, on the electronic ground state PES, early *cis*. 3 ps, green structure: twisted *cis* structure 3 ps after excitation (electronic ground state). I_T_/pR_0_: earliest intermediate determined at the synchrotron. pR_1_ and I_CT_ branch away from I_T_. I_CT_ decays to pR_2_. pR_1_ and pR_2_ finally convert to pB_1_. I_0_ and I_0_^#^: associated spectral intermediates. The conical intersection is shown in yellow, and approximate torsional angles are reported (ϕ_tail_). Important chromophore pocket residues are marked; hydrogen bonds are indicated by dashed lines.

The interest then shifted to intermediates that are populated on time-scales from 100 ps to 10 ns. The fastest time delay is set by the 100 ps pulse duration at the synchrotron. In 2013, the structure of an early intermediate I_T_ (T for twisted)on time scales <1 ns was published.^2^ The chromophore is half-way *cis*. This structure is considered in transition from *trans* to *cis*. However, another group determined that this chromophore structure is already almost *cis*.^27^ The structure was called pR_0_. pR_0_ and I_T_ are almost identical, but differ in the torsional angle of the chromophore tail (Fig. 4, the more *cis*-like 40° for pR_0_ and 90° for I_T_). Due to the lack of experimental restraints on the ps time scale, it is difficult to resolve this discrepancy^48,49^ until atomic resolution X-ray data are available. An experiment at a free electron laser such as the Linac Coherent Light Source (LCLS, see further below) is conceivable that utilizes somewhat larger microcrystals on the order of 50 *μ*m to extend the resolution beyond 1.45 Å, the highest resolution reached for PYP at the LCLS to date.^3^ Perhaps, this experiment could be done using newest fixed target technology, where microcrystals are locked into a regular array of small depressions, which can be quickly scanned through the X-ray beam.^50^ The structure of the intermediate following I_T_/pR_0_, called I_CP_ previously, was found to be bent behind the chromophore plane of the dark state, and the notion of planarity was given up in favor of a bent, twisted structure. I_CP_ was renamed as I_CT_ (*cis*-twisted). Further, pR_E46Q_ already appeared very early on, right after I_T_ decayed. Consequently, pR_E46Q_ was renamed as pR_1_. Finally, structure pR_cw_ that appeared later than I_CP_ (now I_CT_) is renamed as pR_2_. The early part of the photocycle now looks like the one shown in Fig. 3(a). Three intermediates, I_T_, pR_1_ and I_CT_, contributed to the single 1 ns time point collected in 1998 interpreting the difference electron density features in their entirety.

Near the end of the first decade of the new millennium, the only Laue beamline in the US, BioCARS 14-IDB at the Advanced Photon Source (Argonne National Laboratory) enjoyed a major upgrade.^51^ Laue data could now be collected in a fully automatic way. X-ray beamsizes as small 90 *μ*m (h) × 60 *μ*m (v), much smaller than the crystal size, were employed. Control experiments made sure that the kinetics did not change due to the intense ionizing X-ray radiation. A new dose limit, the kinetic dose limit D_K_^1/2^ was determined for PYP. Below the D_K_^1/2^, the kinetics does not change. For PYP, D_K_^1/2^ is only slightly smaller than the conventional dose limit D_1/2_^52,53^ which characterizes structural damage. It became possible to collect an entire time series consisting of up to 30 time points^54^ from a single PYP crystal without exceeding D_K_^1/2^. The collection of a time series at ambient temperatures, for example at 20 °C, only takes on the order of 5 h assuming 20 crystal settings to cover reciprocal space, 7 exposures/diffraction pattern, 30 time points, and 4 s wait between the pump-probe sequences to allow the full completion of the photo-reaction. This provided the opportunity to change an additional parameter, the most important of which is the temperature. Chemical reactions in general are temperature activated and their temperature dependences can be followed by crystallography.^55^ The PYP photocycle was investigated with 14 different temperature settings from −40 °C to 70 °C.^56^ At −40° C, the one end of the temperature range, the photocycle takes several 10 s to complete, and by setting the temperature slightly higher than 70 °C, the other end of the temperature range, crystals quickly deteriorate. However, within this range meaningful, comprehensive time series can be collected. A new type of crystallography emerges, five-dimensional macromolecular crystallography,^56,57^ which enables the determination of barriers of activation by changing the temperature in addition to the 4 other variables, space and time. At −40 °C, it takes approximately 1.5 days of synchrotron time to collect the time-series, at 70 °C a time series can be collected as short as in 2 h. The SVD based analysis was of central importance to globally determine the relevant kinetic phases in the temperature dependent time series of difference maps. All time-series were analyzed with a mechanism similar (but not identical) to that shown in Fig. 3(a) employing the intermediate states and their corresponding structures described above. By successively increasing the temperature, the photocycle accelerates by a factor of about 500, goes through a maximum, and slows down again above 50 °C, which represents the temperature maximum of the biological, macromolecular reaction. In Fig. 3(b), the temperature dependence of one of the rate coefficients (k_4_) in the mechanism is shown. The exponential dependence can be fitted with the transition state theory,^58^ from with enthalpy and entropy differences to the transition state can be determined. These values can then be compared with values found in solution.^45^ For rate k_4_, results from crystallography and solution agree sufficiently. However, for rate k_6_ (the pB to pG transition), the situation is different. The entropy difference to the transition state is very negative in solution (−196 J mol^−1^ K^−1^)^45^ whereas a small positive value is found in the crystals (16 J mol^−1^ K^−1^).^56^ This corroborates the view that in solution the recovery of the ground state starts from a comparatively much more disordered pB state^44,46^ that must refold back to pG through a better ordered transition state. Since the kinetics is temperature activated, early intermediates that decay faster than the time-resolution at room temperature can be observed by decreasing the temperature. At −30 °C, intermediate I_T_, which decays within 1 ns at room temperature and could only be observed with sub-ns time resolution, can be conveniently observed up to 10 ns.^56^

Synchrotron based experiments on PYP were all concluded in 2013 by pushing the time resolution to the 100 ps pulse duration limit. 4 years earlier (in 2009) the LCLS at Stanford Linear Accelerator Center (SLAC), the world's first free electron laser came online and allowed time-resolutions as short as a few femtoseconds, the pulse duration of the free electron lasers. One particular way to collect X-ray data at the XFEL is to use the method of serial femtosecond crystallography (SFX).^59^ To conduct a time-resolved SFX experiment on PYP, microcrystals^60^ must be grown and a suitable laser setup is necessary which must be synchronized to the experiment. In order to reach the femtosecond time scale, two proofs of principle experiments were necessary. First, it needed to be established that time resolved SFX experiments are indeed feasible at the LCLS with near atomic resolution, and second, it had to be established that the photocycle can be started with femtosecond laser pulses. Both control experiments were successfully conducted.^61,62^ It became clear that the extent of photoinitiation is much larger with microcrystals compared to macrocrystals. With nanosecond laser pulses, yields as high as 40% were reached. In comparison with macroscopic crystals at the synchrotron, the yield is only 10% in favorable cases. The reason is that the penetration depth is small, about 20 *μ*m, even if laser wavelengths that substantially deviate from the absorption maximum are used. Starting a reaction by wavelengths close to the absorption maximum seems to be practically difficult because the thin layer on the surface which is activated and the much larger X-ray beam size at the synchrotron do not match. However, with 5 *μ*m crystals, the penetration depth at the absorption maximum and crystals size match which allows for optimal reaction initiation not only with ns laser pulses but also with fs pulses. The advantage of ns pulses is that those molecules that initially and very rapidly revert to the dark state can be excited anew, multiple times, which boosts the apparent photoactivation yield. Femtosecond pulses are needed to reach fs time resolution. However, the molecules are excited only once, the apparent yield is the primary yield, and a much smaller extent of photoactivation is expected. It is essential in this case to photoactivate the crystal as optimally as possible.^63^ In addition, care has to be taken to limit the laser intensity to avoid non-linear processes (two-photon absorption). The intensity must be selected so that an optimal population of excited molecules on the electronic excited state potential energy surface (PES) S_1_ is achieved.^63,64^ A quick calculation can be made by matching the number of photons to the number of PYP molecules illuminated. However, a better way is to perform a spectroscopic experiment on a micrometer thick crystalline layer, which was achieved by crushing a macroscopically large crystal between two coverslides.^64,65^ These experiments provide a limit on the acceptable intensity and also give information about damage and the extent of non-linear processes when the laser intensity is increased beyond this limit.^64^

The time-resolved crystallographic experiments with ultrafast time resolution were conducted at the LCLS in March 2015 with 300 fs, 600 fs, and 3 ps nominal time delay settings.^62^ The jitter of the SASE X-ray pulses relative to the laser pulses as well as drifts away from the nominal settings was measured on the shot by shot basis using a time tool developed at the LCLS.^66^ With the time tool, the jitter was determined within about 100 fs. Drift and jitter distributed the nominal settings through a wide range of actual time-delays, which were collected into time bins spanning from 140 fs to 1000 fs. No jitter correction was performed for the 3 ps time-delay. The *trans* to *cis* isomerization occurs within 600 fs (Fig. 4). 140 fs after excitation the chromophore has moved already quite substantially, but is still trans with a torsional angle of 150°. The tail bends behind the position of the double bond in the reference structure. This is a structure ready to isomerize. At 800 fs, the torsional angle is on the order of 60°. This is already very close to the 40° observed after 3 ps. Small structural rearrangements occur between 800 fs and 3 ps. The nature of this transition has been examined in 2004 by computer simulations^67^ and its time scale has been also experimentally confirmed by spectroscopy.^21,68^ Most interestingly, the *trans* to *cis* isomerization occurs at the conical intersection seam which connects the electronic excited state PES with the electronic ground state PES.^67^ The transition through the conical intersection has been now observed and characterized structurally with time-resolved crystallography in real time (Fig. 4). Difference maps on the 800–1000 fs time scale are quite noisy, which might indicate the strong release of stored energy as heat after the transition. After 3 ps (and actually also before the transition), the molecules in the crystals are comparatively cool and clearer difference maps are observed. The structure of the 3 ps time point is essentially identical to the structure of I_T_/pR_0_. The torsional angle observed favors the near-*cis* torsional angle of pR_0_. It is very likely that, in contrast to the later events in the photocycle, the very early processes up to a few ps are not temperature activated and cannot be trapped at cryogenic conditions. The ultrafast dynamics should therefore be temperature independent, mainly driven by electrostatic forces^62^ on the respective energy surfaces, and promoted by specific modes.^68^ Time resolved crystallography at the free electron laser completed the view of the PYP photoreaction in a sense that it provided the structural base for the *trans* to *cis* isomerization and the photocycle that follows. However, the time scale faster than 100 fs has not been time resolved. An instantaneous process is observed within the transition from the ground state PES to the excited state PES (S_0_ to S_1_ transition) and the first few femtoseconds after this. With few-fs or attosecond X-ray sources that are currently under construction these processes should become observable.

In summary, more than 25 years of structure determination on the PYP photocycle enabled for the first time the determination of a complete structural view of the chemically so important *trans* to *cis* isomerization in a biomolecule, starting with fundamental motions on the femtosecond time scale and ending with barriers of activation of the slowest processes in the kinetic mechanism. In addition, PYP has been a model system employed for the development of numerous data collection techniques and computer algorithms. We have learned so much from this small protein, and we are continuing to do so with the advent of attosecond X-ray sources. PYP crystals are superb and will allow us to test these new sources and derive meaningful results from them. PYP is a balancing act between biology, chemistry, and physics contributing to light perception, molecular orbital theory, and finally atomic physics on the attosecond time scales. It has rarely been such a versatile molecule capable of covering such a wide range of applications. The time was well invested.
